# Facilitators and barriers of reducing sedentary behavior in sedentary and non-sedentary older adults: a descriptive qualitative study based on the COM-B model and TDF

**DOI:** 10.1186/s12889-025-23613-3

**Published:** 2025-07-16

**Authors:** Siqing Chen, Kaijie Yang, Albert Ko, Edward Giovannucci, Matthew Stults-Kolehmainen, Lili Yang

**Affiliations:** 1 Nursing Department, Sir Run Run Shaw Hospital, Zhejiang University School of Medicine, Hangzhou, China; 2 Department of Epidemiology, Harvard T.H. Chan School of Public Health, Boston, MA USA; 3 Department of Biobehavioral Sciences, Teachers College, Columbia University, New York, NY USA; 4 School of Medicine, Zhejiang University, Hangzhou, Zhejiang China; 5 Center for Weight Management, Digestive Health Multispecialty Clinic, Yale New Haven Hospital, New Haven, CT USA

**Keywords:** Sedentary behavior, Barriers, Older adults, Facilitators, COM-B, TDF, Mobile health

## Abstract

**Background:**

Prolonged sedentary behavior is a critical health risk for older adults. However, little is known about the distinct barriers and facilitators experienced by sedentary and non-sedentary older adults. Understanding these factors is essential for designing effective behavior change interventions.

**Purpose:**

The study aims to identify and categorize the barriers and facilitators to reducing sedentary behavior among sedentary and non-sedentary older adults using the Capability, Opportunity, Motivation-Behavior (COM-B) model and Theoretical Domains Framework (TDF), thereby informing future mobile health (mHealth) interventions designed to reduce sedentary time in this population.

**Methods:**

Data were collected through semi-structured interviews with older adults, conducted at two community hospitals in China between July 2024 and September 2024. The interviews focused on older adults’ psychological and physical capabilities, social and physical opportunities, and reflective and autonomous motivations related to sedentary behavior. According to the Canadian 24-Hour Movement Guidelines, participants were classified as sedentary (> 8 h/day sitting time) or non-sedentary (≤ 8 h/day) based on a participant characteristics questionnaire with verbal confirmation during the interview. The data were analyzed thematically, and the identified themes were mapped onto the COM-B model and TDF. Study procedures followed the COREQ checklist for qualitative research reporting.

**Results:**

The study included 29 older adults, comprising 19 sedentary (65.5%) and 10 non-sedentary (34.5%). The following ten higher-order themes were identified: Lack of Knowledge (and Limited Knowledge); Lack of Methods (and Available Methods); Sedentary Triggers (and Interruptions); Lack of Management (and Self-management); Lack of Social Support (and Available Social Support); Lack of Environmental Support (and Available Environment Support); Perceptions and Conflicts (and Importance and Effort); Lack of Confidence (and Confidence); Limited Belief (and Understanding Health Benefits); and Limited Motivation (and Sufficient Motivation).

**Conclusion:**

Sedentary older adults face barriers such as low awareness of health risks, lack of regulation strategies, and insufficient social support, while non-sedentary older adults demonstrate higher confidence, better self-regulation, and engage in structured activities supported by cues such as mobile health reminders.

**Supplementary Information:**

The online version contains supplementary material available at 10.1186/s12889-025-23613-3.

## Introduction

Sedentary behavior refers to any waking activity characterized by an energy expenditure of 1.5 Metabolic equivalents (METs) or less while in a sitting, reclining, or lying position [1]. In recent years, sedentary behavior among older adults has garnered significant attention due to its widespread prevalence [2]. Older adults with high levels of inactivity and low levels of physical activity (not meeting WHO’s 150-minute weekly moderate activity guideline) are especially vulnerable to the adverse effects of prolonged sedentary behavior. A global survey revealed that adults aged 60 or older spend an average of 9.4 h per day sedentary, accounting for 65–80% of their waking hours [3]. Such behavior is closely associated with an increased risk of chronic diseases like obesity, diabetes [2], and cardiovascular diseases [4, 5], and other severe health conditions, including cancer [6], dementia [7, 8], accelerated aging [9], and higher all-cause mortality [10]. Despite the growing awareness of these health risks, including the release of the “2020 WHO Guidelines on Physical Activity and Sedentary Behavior” [11] and the Canadian 24-hour Movement Guidelines (recommending ≤ 8 h/day sedentary time) for older adults [12], many in this population face persistent challenges in achieving recommended reductions in sedentary time [13, 14]. The underlying reasons for this phenomenon are complex and multifaceted, encompassing capabilities, environmental opportunities, and intrinsic and extrinsic motivations [15]. Based on this, how to effectively identify and address these factors to reduce sedentary behavior among older adults has become an urgent study issue.

The Capability, Opportunity, Motivation-Behavior (COM-B) model posits that human behavior emerges from the interaction of three core components: capability (one’s physical and psychological capacity to act), opportunity (available environmental and social resources), and motivation (both reflective and automatic drivers of behavior). Building on this foundation, the Theoretical Domains Framework (TDF) translates these components into 14 practical domains, including knowledge, social influences, and environmental context, providing a systematic approach to identifying and addressing behavioral determinants [16]. The COM-B model explains behavior formation through capability, opportunity, and motivation, while the TDF further refines these dimensions, providing a more comprehensive perspective for examining the possibilities of behavior change [16] through in-depth interviews and analysis of sedentary behavior among older adults. To better understand the factors influencing sedentary behavior in older adults and explore potential intervention strategies, this study applies the COM-B model and the TDF to analyze sedentary behavior among older adults. While these frameworks have been used to develop sedentary behavior interventions for occupational groups [17, 18], research focusing on specific populations, such as older adults remains limited. Understanding the factors that influence sedentary behavior is crucial, as it empowers participants to actively engage in the decision-making process for behavior change [19].

Mobile Health (mHealth), defined as the use of mobile devices (e.g., smartphones, wearables) to deliver health-related services and behavior change support, has emerged as a promising approach to address sedentary behavior due to its accessibility, scalability, and potential for personalized feedback [13]. For older adults, mHealth interventions can overcome traditional barriers such as limited mobility, geographic isolation, or lack of in-person resources [13, 20, 21]. Furthermore, Behavior change techniques (BCTs) are a systematic set of 93 strategies that can be utilized to develop and implement various mHealth interventions for sedentary behavior [16]. Studies have shown [22, 23] that mHealth-based interventions incorporating BCTs (e.g., self-monitoring, goal setting, prompts) potentially reduce sitting time in older adults, highlighting its potential as a scalable solution.

Therefore, this study aims to identify key barriers and facilitators to reducing sedentary behavior among sedentary and non-sedentary older adults, and to examine how these factors manifest across different dimensions. By systematically analyzing interview data, and deepening the understanding of population-specific challenges and motivators, thereby informing the development of tailored mHealth interventions.

## Methods

### Study design

A descriptive qualitative study design was employed to gain in-depth insights into the factors influencing sedentary behavior among older adults. This approach allowed for a detailed exploration of older adults’ psychological and physical capabilities, social and physical opportunities, and reflective and autonomous motivations related to sedentary behavior through semi-structured interviews conducted between July 2024 and September 2024. Consolidated Criteria for Reporting Qualitative Research (COREQ) checklist was used in this study to report the findings [24].

### Theoretical framework

**Fig. 1:**
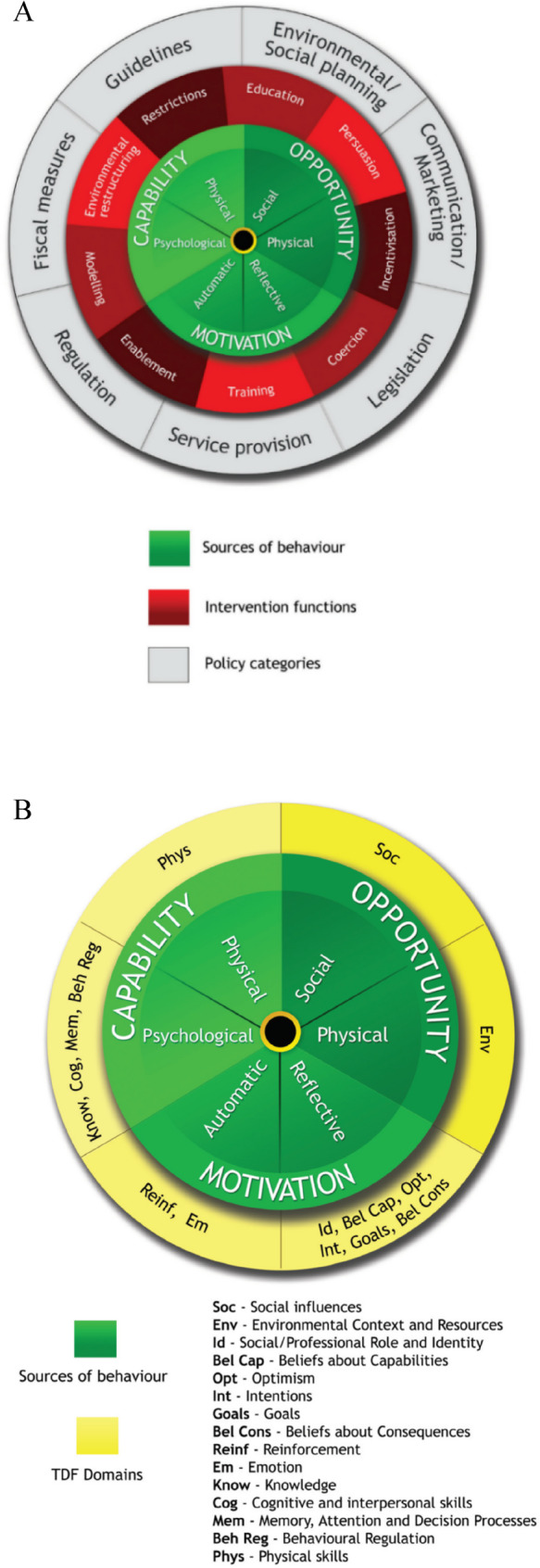
COM-B model (**A**) and TDF framework (**B**)

The Medical Research Council recommends that researchers developing complex interventions should have a theoretical understanding of the change process, which can be informed by existing evidence, theoretical frameworks, and, where necessary, qualitative interviews [25, 26]. The COM-B model and the TDF will guide the qualitative analysis of factors influencing sedentary behavior among older adults. With its core component, the COM-B model, identifies Capability, Opportunity, and Motivation, as the essential elements driving behavior. These three factors interact dynamically to determine whether a behavior can be initiated or sustained. The COM-B model has been successfully applied in various contexts, such as occupational sedentary behavior, physical activity, smoking cessation, stroke rehabilitation and diabetes prevention [16, 17, 27]. To better understand the behavior change process, the TDF is used to systematically analyze behavioral determinants. The TDF encompasses 14 domains and is often combined with the COM-B model to provide a more detailed analysis of factors influencing behavior (Fig. 1A, B) [28, 29] (Table 1).

### Setting

This study was conducted in two community hospitals in Hangzhou, Zhejiang Province, China.

### Participants

Older adults were recruited using a purposive sampling method. Inclusion criteria for this study included: (a) aged 60 years or older; (b) able to communicate effectively in Mandarin or local dialect; and (c) capable of providing informed consent. All older adults recruited were willing to participate in the interviews. Participant characteristics (e.g., age, gender, marital status) were collected through a structured questionnaire administered prior to the interviews. Older adults were categorized as sedentary or non-sedentary based on self-reported sitting time collected through a demographic characteristics questionnaire and confirmed verbally during the interview. Sedentary classification was assigned to those reporting > 8 h/day of sitting time, consistent with the Canadian 24-Hour Movement Guidelines for Adults [12], while non-sedentary participants reported ≤ 8 h/day.

### Data collection

This study utilized semi-structured, face-to-face in-depth interviews as the primary method for data collection, which is suitable for exploring older adults’ perceptions and opinions on sedentary behavior. To ensure the objectivity, trustworthiness, and richness of the collected data, the interview guide was systematically developed based on the COM-B model and the TDF. The guide specifically targeted the psychological and physical capabilities, social and physical opportunities, and reflective and autonomous motivations of sedentary behavior, ensuring a comprehensive understanding of barriers and facilitators of reducing sedentary behavior. Detailed components of the interview guide are provided in Table 1.

All interviews were conducted by a Ph.D. candidate (SC) and a specialist nurse in geriatric care (DS), both trained in qualitative study and semi-structured interviewing techniques. To maintain consistency, the investigator and older adults were unknown to each other before the interviews, which were conducted in quiet settings, such as community centers. Field notes were taken during the interviews to capture contextual details. The duration of the interviews ranged from 15 to 30 min. The audio recordings were transcribed verbatim by the researcher within 24 h of each interview to ensure data accuracy in the data. The transcriptions were then analyzed, and data saturation was assessed as each transcript was reviewed. The interviews continued until no new significant information emerged, ensuring data saturation was achieved [30]. The data collection process spanned from July 2024 to September 2024.

**Table 1:** Interview guide

COM-B Model	TDF	Questions
**Psychological Capability**	Knowledge	What do you know about sedentary behavior? Could you describe your understanding of sedentary behavior in detail?
	Behavioral Regulation	If you sit for an extended period, do you use any apps or methods to interrupt your sedentary behavior?
	Memory, Attention, and Decision Processes	What are the reasons that lead you to sit for extended periods? Do you use any prompts?
**Physical Capability**	Skills	How much do you know about sedentary behavior? What skills do you think are required to reduce sedentary behavior? Do you need behavioral counseling for sedentary reduction?
**Social Opportunity**	Social Influence	How do your friends or others help or hinder your efforts to reduce sedentary behavior?
**Physical Opportunity**	Environmental and Resource Factors	What work or home environmental factors support or hinder your efforts to reduce sedentary behavior?
**Reflective Motivation**	Social/Professional Roles and Identity	To what extent do you consider reducing sedentary behavior to be part of your social role? Why do you think reducing sedentary behavior is important for you?
	Belief in Capabilities	How confident are you in your ability to reduce sedentary behavior? What factors increase or decrease your confidence?
	Belief in Consequences	What do you think are the benefits of reducing sedentary behavior? What specific benefits do you think it could bring?
**Automatic Motivation**	Optimism	What positive outcomes do you think reducing sedentary behavior could bring? How confident are you in achieving these outcomes?
	Intention	Do you plan or intend to reduce sedentary behavior? Why or why not?
	Goals	When reducing sedentary behavior, what are your goals?
	Reinforcement	What do you think motivates you to reduce sedentary behavior?
	Emotions	How do you think reducing sedentary behavior would make you feel?

### Data analysis

Inductive thematic analysis was employed for the data analysis. Thematic analysis is a method used to identify, analyze, organize, describe, and report themes within a dataset [24]. Three coders, two Ph.D. students (SQC, JKY) and a master’s student (AK), all with strong backgrounds in qualitative research and thematic analysis, had received formal instruction in qualitative study methods before conducting this study, and each independently coded the transcripts. Initial codes were synthesized into lower-order themes (LOTs) for sedentary and non-sedentary older adults, which were cross-checked by the coders to ensure alignment with the data. Subsequently, these LOTs were systematically organized into higher-order themes (HOTs) by mapping them to the COM-B model and the Theoretical Domains Framework (TDF), capturing the psychological and physical capabilities, social and physical opportunities, and reflective and automatic motivations underlying sedentary behavior among older adults. Conflicting codes were discussed among the coders (SC, JY, AK) and, if necessary, resolved with senior researchers (LY and EG) to reach a consensus. To ensure the credibility of the findings, we adopted the criteria proposed by Lincoln and Guba [31], focusing on credibility, transferability, dependability, and confirmability. Transferability was supported by providing detailed descriptions of the study settings and older adults, allowing readers to assess the applicability of findings to other contexts. QRS NVivo software (version 14) was utilized for data management and to assist in the coding process.

### Ethics approval and consent to participate

Ethical approval for this study was granted by the Ethics Committee of Zhejiang University (K2023150). The study was conducted in accordance with the ethical principles outlined in the Declaration of Helsinki. Written informed consent was obtained from all participating older adults prior to their inclusion in the study. The consent process included detailed information about the study purpose, procedures, potential risks and benefits, confidentiality measures, the voluntary nature of participation, and the right to withdraw at any time. Participants also provided explicit permission for the publication of anonymized findings derived from their responses. Strict confidentiality was maintained throughout the research process.

## Results

A total of 29 older adults participated in this study, comprising 11 males and 18 females, aged between 60 and 73 years, predominantly married older adults. They lived in villages, towns, or cities, either with their spouse, children, or alone. Educational levels ranged from primary school to graduate degrees, with the majority having a middle or high school education. Employment statuses varied; most older adults were retired, some were self-employed, and a few were unemployed or actively working. Health insurance types included medical insurance or self-paid, and common health conditions were hypertension, hyperlipidemia, hyperglycemia, and chronic diseases. Table 2 provides a comprehensive overview of the older adults’ demographic characteristics.

Older adults were categorized as sedentary (*n* = 19) or non-sedentary (*n* = 10). We identified 94 and 54 lower-order themes (LOTs) among sedentary and non-sedentary participants respectively. The following ten higher-order themes were identified: Lack of Knowledge (and Limited Knowledge); Lack of Methods (and Available Methods); Sedentary Triggers (and Interruptions); Lack of Management (and Self-management); Lack of Social Support (and Available Social Support); Lack of Environmental Support (and Available Environment Support); Perceptions and Conflicts (and Importance and Effort); Lack of Confidence (and Confidence); Limited Belief (and Understanding Health Benefits); and Limited Motivation (and Sufficient Motivation). Comprehensive details regarding both barrier and facilitator factors are provided in Supplementary Material 1.

**Table 2:** Participant characteristics (*n* = 29)

Participant	Age (years)	Gender	Marital status	Current residence	Household members	Education level	Employment status	Insurance type	Current/Past medical history	Limit mobility	Sedentary preference
S1	65	Female	Married	Village	Children	Primary School	Retired	Self-Paid	None	No	Yes
S2	61	Male	Married	Town	Spouse	Associate Degree	Construction	Medical Insurance	Not Mentioned	No	Yes
S3	62	Female	Married	City	Spouse	High School	Retired	Medical Insurance	Chronic Disease, Hyperlipidemia	No	Yes
S4	70	Female	Married	Village	Spouse	Primary School	Retired	Self-Paid	Not Mentioned	No	Yes
S5	61	Male	Married	Town	Spouse	Middle School	Self-Employed	Self-Paid	Not Mentioned	No	Yes
S6	60	Female	Married	Town	Spouse	Associate Degree	Retired	Medical Insurance	Not Mentioned	No	Yes
S7	68	Male	Married	Town	Spouse	Middle School	Retired	Self-Paid	Not Mentioned	No	Yes
S8	63	Female	Married	Village	Spouse	Primary School	Self-Employed	Self-Paid	Not Mentioned	No	Yes
S9	63	Male	Married	Village	Spouse/Children	High School	Self-Employed	Self-Paid	Hyperlipidemia	No	Yes
S10	72	Female	Married	Town	Children	None	Unemployed	Medical Insurance	Hypertension	No	Yes
S11	60	Male	Married	Town	Spouse	High School	Self-Employed	Medical Insurance	Hypertension	No	Yes
S12	62	Female	Married	Town	Spouse	Middle School	Tailor	Self-Paid	Hypertension	No	Yes
S13	67	Female	Married	Town	Spouse	None	Retired	Medical Insurance	Hypertension, Hyperthyroidism	No	Yes
S14	60	Female	Divorced	City	None	Bachelor’s Degree	Retired	Medical Insurance	Hypertension	No	Yes
S15	68	Male	Divorced	City	None	Graduate Degree	Foreign Trade Manager	Medical Insurance	Hypertension, Hyperglycemia	No	Yes
S16	62	Male	Married	City	Spouse	Bachelor’s Degree	Active Teacher	Medical Insurance	Hypertension, Hyperglycemia, Fatty Liver	No	Yes
S17	62	Female	Married	City	Spouse	High School	Self-Employed	Self-Paid	Hypertension, Hyperglycemia, Breast Cancer	No	Yes
S18	66	Female	Married	City	Spouse	High School	Retired	Medical Insurance	Hyperlipidemia	No	Yes
S19	64	Male	Divorced	City	None	High School	Retired	Medical Insurance	Cervical Spondylosis, Lumbar Spondylosis	No	Yes
N1	73	Female	Married	Town	None	Primary School	Unemployed	Self-Paid	Not Mentioned	No	No
N2	67	Female	Married	Town	None	Middle School	Retired	Medical Insurance	Chronic Disease, Diabetes, Hypertension	No	No
N3	68	Female	Married	Town	Spouse	High School	Retired	Medical Insurance	History of Breast Tumor Surgery	No	No
N4	65	Female	Divorced	Town	Children	Middle School	Unemployed	Self-Paid	Not Mentioned	No	No
N5	60	Female	Married	Town	Spouse	High School	Retired	Medical Insurance	Not Mentioned	No	No
N6	63	Male	Married	Town	Spouse	Middle School	Retired	Medical Insurance	Not Mentioned	No	No
N7	67	Female	Married	Town	None	Middle School	Unemployed	Self-Paid	Not Mentioned	No	No
N8	65	Male	Married	Village	Spouse	Middle School	Retired	Medical Insurance	Not Mentioned	No	No
N9	62	Female	Married	Town	Spouse	Primary School	Retired	Medical Insurance	Cervical Spondylosis	No	No
N10	67	Male	Married	City	Spouse	Vocational School	Retired	Medical Insurance	HyperlipidemiaHyperglycemia	No	No

*S* Sedentary older adults, *N* Non-sedentary older adults

### Capability (psychological capability)

#### Knowledge

Among sedentary older adults, there was a significant lack of knowledge about sedentary behavior. Many older adults could not clearly define what sedentary behavior entailed, often providing vague or incomplete explanations. Some older adults focused only on sitting, while others described it as duration, such as “sitting for long periods.” A few older adults showed slightly more understanding, equating sedentary behavior with being motionless, such as “sitting or lying down without moving.” However, many older adults admitted to not understanding the concept at all. Select quotes from older adults are italicized below:

*I don’t know about sedentary behavior. (S7)*

Non-sedentary older adults demonstrated a generally better understanding of sedentary behavior than sedentary older adults. These individuals displayed a basic awareness of sedentary behavior, whereas very few did not understand the concept. Among those with knowledge, one older adult provided a more detailed explanation, identifying specific risks, such as its impact on energy flow and physical health.

*Sitting for a long time affected the body’ s meridians. (N6)*

#### Behavioral regulation

A lack of effective methods to interrupt sedentary behavior was common among sedentary older adults. Many relied on hunger, urgent tasks, entertainment activities and physiological responses to initiate movement, while some older adults demonstrated spontaneous interruptions, such as standing up after long periods of sitting. However, sedentary older adults did not report using electronic prompts to break their sedentary behavior.

*There’s no method. When you’re hungry*,* it’s time to eat*,* you feel like*,* oh no*,* I have to get up to eat. (S19)*

Non-sedentary older adults tend to interrupt sedentary behavior through spontaneous actions, such as standing up after prolonged sitting. Some older adults mentioned incorporating daily walking routines and using smart devices to prompt breaks and encourage movement.

*Walking*,* I walk tens of thousands of steps every day. (N5)*

#### Memory, attention, and decision processes

Sedentary older adults encountered multiple barriers to reducing their prolonged sitting habits. For the majority, leisure activities, such as playing mahjong, watching television, or engaging in handicrafts, were the primary contributors to extended periods of inactivity. Work-related demands, such as drawing or other desk-based tasks, and ingrained habits like a preference for inactivity, were also significant barriers to reducing prolonged sitting. For a smaller portion of older adults, religious practices, physical limitations, and aging further exacerbated sedentary behavior, as reduced mobility and discomfort led to an increased reliance on sitting and resting. Moreover, the absence of structured reminders or external prompts to interrupt sedentary behavior was a significant barrier for sedentary older adults.

*No (reminders). (S4)*

Non-sedentary older adults demonstrated fewer barriers to maintaining activity and displayed a more balanced approach to breaking sedentary behavior. Leisure activities, such as playing mahjong, occasionally led to prolonged sitting, though these periods were typically shorter and less frequent. Fatigue was another factor contributing to sitting behavior, as some older adults opted to rest when tired. Certain older adults relied on external aids to assist in breaking sedentary behavior, with phone reminders serving as an effective tool.

*If I’ve been looking at my phone for too long*,* it automatically reminds me*,* “You need to rest*,* you need to rest!” (N9)*

### Capability (psychological capability)

Sedentary older adults demonstrated a lack of understanding and awareness of effective strategies to manage sedentary behavior, with limited knowledge of methods for improvement. Their behavior was largely influenced by task demands, and while physical activity or parent-child interactions occasionally interrupted sedentary time, they regarded behavioral counseling for sedentary reduction as unnecessary and irrelevant, often citing time constraints or limited access to structured opportunities as additional barriers to reducing sedentary behavior. However, a minority acknowledged the potential benefits of behavioral counseling for sedentary reduction and expressed openness to learning strategies for reducing sedentary behavior.

*I don’t have (any methods to improve sedentary behavior). (S1)*

In contrast, non-sedentary older adults recognized the negative impact of sedentary behavior on health. Among the reported skills, some older adults described task-driven actions, such as engaging in physical activity when prompted by specific tasks or responsibilities. They displayed a slightly better understanding of strategies for reducing sedentary behavior. Many recognized the importance of breaking long periods of sitting and implemented simple yet intentional strategies, such as standing after extended sitting or incorporating walking into their daily routines. Their attitudes were more mixed: while some felt self-reminders were sufficient and saw little need for formal behavioral counseling for sedentary reduction, others remained uncertain about the potential value of formal counseling.

*Stand more*,* sit less. (N6)*

### Opportunity (social opportunity and physical opportunity)

Sedentary older adults experienced certain limitations in terms of social support. Some older adults lacked clear social encouragement, while others mentioned receiving occasional reminders or support from those around them, such as family members (e.g., sons or partners), to promote activity. A few older adults noted that friends or family members sometimes tried to involve them in social activities. Many sedentary older adults pointed out that work and environmental conditions made it challenging to interrupt sedentary behavior. For instance, some older adults mentioned that their work required prolonged sitting or that their environment was not conducive to frequent movement. Others highlighted how their home environment could reinforce sedentary behavior, such as habitual sitting post-retirement. However, some older adults did not perceive external factors as barriers to reducing sedentary behavior and even mentioned that family support or engaging in activities like childcare could help them break sedentary patterns.

*I don’t have any social or environmental support. (S5)*

In contrast, non-sedentary older adults exhibited more positive characteristics regarding social support. They often referenced explicit encouragement from family members or companions, such as reminders to avoid prolonged sitting or engaging in social activities with friends. However, some non-sedentary older adults reported receiving no external help or support and instead relied entirely on their self-awareness to adjust their behavior. Regarding environmental and resource factors, non-sedentary older adults demonstrated greater adaptability. While some cited external conditions, such as weather, as obstacles to activity, they often found ways to interrupt sedentary behavior through daily tasks, household chores, or outdoor activities. Some older adults mentioned housework, taking care of children, or grocery shopping as effective means to avoid prolonged sitting. Moreover, retirement status was seen as an opportunity rather than a barrier, with non-sedentary older adults leveraging their flexible schedules to incorporate regular physical activity and achieve higher levels of movement.

*I have been retired for 17 years*,* doing housework*,* cooking (improving sedentary behavior) and living alone. (N2)*

### Motivation (reflective motivation)

Many sedentary older adults acknowledged the importance of reducing prolonged sitting, but their understanding of its significance varied. Some older adults recognized it as important for health but were unsure of its full impact. Others emphasized the importance of interrupting sedentary behavior but found it difficult to change due to ingrained habits or external constraints. For some, sedentary behavior had not yet become a fundamental part of their identity, but they still felt that making changes was impossible, expressing a sense of resignation and lack of control over their behavior. Regarding self-efficacy, many lacked confidence in their ability to reduce sedentary behavior, often citing barriers to reducing sedentary behavior, such as work demands environments that reinforced sitting.

*(Confidence) doesn’t seem to be high. I can’t avoid sitting. I sit more than I should. (S1)*

In contrast, non-sedentary older adults demonstrated higher levels of motivation and confidence in managing their sedentary behavior. The majority of these older adults recognized the importance of maintaining activity, often framing it as essential for their overall health and well-being, particularly in older age. These older adults often balance activity and rest, integrating short periods of sitting with intentional movement. Many expressed confidence in staying active and cited specific benefits, such as improved overall health, relief from constipation, feeling more at ease when walking, and enhanced neck and lumbar function.

*Improving sedentary behavior is good for health*,* I’m confident*,* and I pay attention to it. (N2)*

### Motivation (automatic motivation)

Many sedentary older adults reported lacking confidence in achieving positive outcomes through reducing sedentary behavior. Nevertheless, some older adults expressed confidence or even high confidence. A few older adults demonstrated complete confidence in the effectiveness of such changes. Most older adults expressed a willingness to reduce sedentary behavior. However, a few indicated a reluctance to change, citing a preference for the perceived comfort of prolonged sitting. Many older adults lacked clear improvement plans. Nonetheless, some older adults had specific objectives, primarily aimed at improving physical health, alongside secondary goals such as reducing sedentary time, increasing physical activity, and achieving emotional stability. Their motivation often stemmed from a desire to maintain physical health or meet the demands of daily activities, such as cooking, moving more to counteract sedentary behavior, and alleviating back pain. Emotionally, sedentary older adults generally reported mild improvements in physical and mental comfort, although some felt that reducing sedentary behavior did not result in significant psychological benefits.

*My son watches me sitting all day at home*,* but I think if I stand too long*,* I need to sit again. I still want to sit. (S1)*

Non-sedentary older adults, on the other hand, exhibited higher levels of confidence and optimism about the benefits of reducing sedentary behavior. They firmly believed that such changes could substantially improve their physical health. These older adults consistently expressed a strong willingness to adopt and maintain behavioral improvements, with clearly defined goals. Some aimed to control blood sugar levels, increase physical activity to maintain health, or reduce the burden of health concerns on family members. They relied not only on established exercise habits but also on motivations, such as improving blood sugar levels and overall health to sustain their efforts. Unlike the sedentary older adults, all but one respondent reported finding the changes effective, with many experiencing greater satisfaction and physical comfort.

*I have this habit*,* after sitting for a while*,* I definitely stand up*,* move my legs*,* and move my knees. I’m definitely concerned about sitting for too long. (N3)*

## Discussion

To our knowledge, this study is the first to systematically explore the barriers and facilitators of reducing sedentary behavior among sedentary and non-sedentary older adults using the COM-B model and the TDF. The findings revealed significant barriers to reducing sedentary behavior across multiple dimensions for sedentary older adults, while non-sedentary older adults exhibited more factors that promoted behavior change. Notable differences were identified between the two populations in psychological capability, physical capability, social opportunities, physical opportunities, reflective motivation, and automatic motivation. These novel insights highlight the multifaceted factors affecting sedentary behavior and offer critical guidance for designing future targeted intervention strategies. Based on previous studies [32, 33], when facilitators that support behavior change are already present among participants, emphasizing them further in the intervention may lead to ceiling effects. To enhance the effectiveness of the intervention, it is more beneficial to concentrate on overcoming the barriers to reducing sedentary behavior. Therefore, this study will mainly focus on barrier-related factors to sedentary behavior reduction.

Our study found that psychological capability deficits among sedentary older adults were primarily characterized by a lack of understanding of sedentary behavior and an absence of structured methods to consistently interrupt prolonged sitting. Many older adults lacked electronic reminders or other systematic strategies to support behavior change. Older adults often deem traditional behavioral counseling for sedentary reduction inaccessible due to time constraints or limited availability, such as residing far from behavioral counseling for sedentary reduction centers. We also found that most older adults engage in sedentary behavior due to activities such as handicrafts, playing Mahjong, watching television, and other forms of entertainment. Factors such as aging, physical limitations, and personal habits also contribute to their prolonged sitting. In contrast, non-sedentary older adults exhibited more substantial psychological capabilities, including a clearer understanding of sedentary behavior and the ability to implement simple yet effective strategies, such as setting smartphone reminders or integrating walking into their daily routines. Ojo et al. [18], emphasized that knowledge is a critical domain within the TDF. Gardner et al. [34] emphasized education, environmental restructuring, and self-monitoring as promising BCTs. By integrating these techniques into mobile apps or wearable devices, older adults can access short videos, audio guides, or text reminders anytime and anywhere to learn about the risks of sedentary behavior and strategies to reduce it. This approach minimizes reliance on in-person behavioral counseling for sedentary reduction, providing a more accessible, personalized, and adaptable intervention for older adults.

Specifically, for psychological capability, knowledge-related BCTs provide information about health outcomes, such as offering sedentary behavior-related knowledge and information on the benefits of reducing sedentary time through the mHealth platform. For behavioral regulation, BCTs such as prompts/cues and self-monitoring can be employed, where the platform provides reminders and allows older adults to track sedentary behavior, including the type, duration, frequency, and/or intensity. For memory, attention, and decision processes, BCTs, such as prompts/decisions can be used, such as setting timed reminders to break sedentary periods and encourage regular activity, helping to create a positive environment for reducing sedentary behavior. The BCTs of goal-setting (behavior) can also be incorporated, where the platform sets goals to reduce sedentary behavior. For physical capability, skills-related BCTs can include behavioral modeling, where the platform offers examples of successful sedentary behavior interruption, demonstrating accurate and standardized techniques for older adults. The BCT of instruction on how to perform behaviors can also be used, to inform older adults about areas needing improvement in reducing sedentary behavior. BCTs, such as behavioral practice/rehearsal, can also be implemented, encouraging older adults to practice and repeatedly apply sedentary behavior interruption strategies.

Furthermore, our study found that social and physical opportunities are also critical external factors influencing sedentary behavior, which aligns with previous findings of Ojo et al. and De Cocker et al. [18, 35]. Sedentary older adults often lack clear social support, and receive minimal reminders or encouragement from family members and friends, with some older adults reporting no social interaction at all. This lack of support reinforces sedentary behavior patterns. Environmental constraints, such as work requiring prolonged sitting or limited space for physical activity at home, further hinder older adults’ behavior change. In contrast, non-sedentary older adults benefit from significantly stronger social support, with family and friends actively participating in maintaining their active behavior. They also demonstrate flexibility in overcoming external challenges, such as weather conditions, by incorporating activities like household chores to maintain higher levels of physical activity.

To address the lack of social and physical opportunities among sedentary older adults, combining the mHealth platform with BCTs focused on social support may be highly effective. Previous studies [22, 36–38] have implemented social support strategies through mHealth platforms to reduce sedentary behavior. These platforms have interactive features that allow participants to share activity logs with family and friends and exchange encouraging messages. The studies mentioned above employed the BCT of emotional support, suggesting that older adults invite family members, friends, or colleagues to regularly break up sedentary behavior together. The BCT of behavior regulation can incorporate social support by offering suggestions on how to regularly interrupt sedentary behavior through the mHealth platform. Additionally, social environment restructuring can encourage older adults to form connections with individuals who are less inclined toward sedentary activities [16]. In cases of bad weather, the mHealth platform can also suggest practical social support strategies, such as engaging in sedentary behavior interruption or indoor exercises.

Inadequate reflective and automatic motivation posed significant challenges for sedentary older adults in changing their behavior. For reflective motivation, sedentary older adults showed varying levels of confidence. Most sedentary older adults lacked confidence in the outcomes of reducing sedentary behavior, doubting whether sedentary behavior change could bring noticeable benefits. By contrast, non-sedentary older adults displayed much higher confidence, firmly believing that reducing sedentary behavior could significantly enhance their health, including improving blood sugar control, reducing joint pain, and increasing vitality. Although sedentary older adults recognized the importance of reducing sedentary behavior, particularly its potential health benefits, many older adults struggled to translate this awareness into action due to a lack of motivation, unclear goals, and ingrained habits. Some older adults admitted that, despite understanding the risks of prolonged sitting, they felt unmotivated or perceived sedentary behavior as unchangeable, often citing external barriers to reducing sedentary behavior like family or work environments.

In contrast, non-sedentary older adults demonstrated stronger reflective and automatic motivation. They had clear goals and a strong belief in the health benefits of reducing sedentary behavior, as well as taking consistent action through strategies such as standing intervals and daily walking. Their motivation was often driven by internal needs, such as relieving constipation, reducing discomfort, and improving longevity, reinforced by their active habits. These findings highlight that enhancing reflective and automatic motivation is essential for encouraging behavior change in sedentary older adults. Based on Zelle et al., increasing self-efficacy by persuading older adults to believe in their ability to perform a behavior and encouraging them to do so is an effective strategy [39]. Therefore, future studies could employ the BCT of commitment, where older adults are encouraged to confirm or reaffirm their commitment to starting, continuing, or resuming regular attempts to reduce sedentary behavior using terms such as “strongly,” “commit,” “high priority,” or “I will.”

Positive reinforcement mechanisms, such as financial rewards or real-time feedback, could further sustain behavior change and enhance a sense of achievement [16]. Studies by Gardner et al. and Lyons et al. [34, 38] emphasize the effectiveness of self-monitoring, goal setting, and economic incentives in promoting sedentary behavior change. Lyons et al. [38] applied the BCT of reward and punishment, such as providing feedback icons and offering financial incentives (participants received $100 after completing assessments). The BCT of health outcome information could be utilized, where the mHealth platform provides information on the benefits of interrupting sedentary behavior via videos. The BCT of credible sources could be employed by presenting videos from healthcare professionals emphasizing the importance of interrupting sedentary behavior. The BCT of prompt/cue could remind older adults to interrupt their sedentary behavior, while The BCT of feedback on behavior could inform them about their activity, such as how many steps they have taken each day. The BCT of self-monitoring could be used to track the type, duration, frequency, and/or intensity of sedentary behavior. The BCT of behavioral modeling could provide examples of older adults regularly interrupting sedentary behavior. The BCT of behavioral contract could be implemented by having older adults sign a contract via the mHealth platform to ensure regular interruption of sedentary behavior. The BCT of goal setting could be used to set specific goals for improving sedentary behavior. The BCT of action planning could assist users in creating a schedule and selecting times to interrupt their sedentary behavior, while the BCT of behavior goal review could allow older adults to check whether their performance aligns with their goals and adjust their future targets accordingly. Additionally, a previous study [40] shows that older adults with anxiety and/or depression tend to be more sedentary and have lower adherence to physical activity. This suggests that healthcare providers could apply the BCT of reducing negative emotions from inadequate autonomy-related motivation by offering guidance on how to manage negative emotions and explaining how interrupting sedentary behavior can help alleviate these emotions.

Although sedentary behavior intervention strategies have been developed for occupational populations [17, 18], studies and practical interventions targeting older adults remain significantly inadequate. Due to their unique physiological characteristics and lifestyles, older adults require more personalized and scientifically tailored intervention approaches [2]. Based on the findings of this study, adopting the COM-B model and the TDF, future studies should prioritize developing mHealth intervention tools tailored for older adults in China. These interventions must not only accurately assess the sedentary behavior patterns of older adults but also provide targeted interventions based on specific sedentary activities, such as watching television or playing mahjong. Furthermore, these interventions should integrate BCTs, such as education, reminders, and goal-setting, to enhance adherence to interventions, enabling older adults to gradually reduce sedentary time. In addition, future studies should focus on evaluating the long-term effectiveness of these interventions, particularly their sustainability and stability in the daily lives of older adults. The design of these interventions should emphasize personalization and usability to ensure high engagement and adherence over time. These interventions should be adaptable and scalable to facilitate their implementation across different regions and cultural contexts, thereby addressing the diverse health needs of older adult populations worldwide. By developing scientifically grounded and technology-driven interventions, it will be possible to provide systematic solutions for managing sedentary behavior in older adults.

### Strengths and limitations

This study offers several key advantages. Firstly, the study applies the COM-B model and the TDF, which are widely recognized frameworks for understanding behavior change. This approach not only facilitates the systematic identification of the psychological and physical capabilities, social and physical opportunities, and reflective and autonomous motivations related to sedentary behavior among older adults but also sheds light on the mechanisms underlying behavior change. Secondly, incorporating both sedentary and non-sedentary older adults provides us with comparative insights into barriers and facilitators of reducing sedentary behavior, which are critical for tailoring interventions. Thirdly, these findings provide valuable information for the development of targeted interventions, particularly mHealth platform, by identifying specific behavioral, environmental, and motivational factors to address. However, this study has certain limitations. Firstly, it focused exclusively on older adults within the Chinese context, potentially limiting the generalizability of the findings to other cultural or geographical settings. To ensure broader applicability, future studies should validate these findings across diverse populations and regions. Secondly, this study captured older adults’ perspectives on sedentary behavior and the decision-making mechanisms influencing their actions. However, it did not include input from healthcare professionals, which could provide valuable insights for practical intervention design.

## Conclusion

Sedentary older adults faced barriers to reducing sedentary behavior, such as low awareness of health risks, lack of regulation strategies, and insufficient social support. In contrast, non-sedentary older adults demonstrated greater confidence, stronger self-regulation abilities, and engagement in structured physical activities, such as regular walking, often supported by cues like mobile health reminders. To address these challenges, a combination of BCTs such as education, self-monitoring, prompts, goal-setting, behavioral modeling, feedback, practice, social support, and environmental restructuring, can be employed to enhance older adults’ capability, opportunity, and motivation to reduce sedentary behavior. Future studies should utilize the COM-B model and the TDF to design targeted mHealth interventions, focusing on identifying the most effective combinations of BCTs to optimize behavior change among older adults.

## Supplementary Information

Supplementary Material 1.

Supplementary Material 2.

## Data Availability

All relevant data are within the manuscript.
